# Dataset and tools for benchmarking multi-sensor multi-people tracking for ground robots

**DOI:** 10.1016/j.dib.2026.112977

**Published:** 2026-06-13

**Authors:** Roberto Larcher, Davide Farina, Marco Piazzola, Franca Corradini, Carlo Grigioni, Jérôme Guzzi

**Affiliations:** a Spindox Labs, Via alla cascata 56/c, 38123 Trento, Italy; b Dalle Molle Institute for Artificial Intelligence Studies (IDSIA USI-SUPSI), Via la Santa 1, CH-6900 Lugano, Switzerland

**Keywords:** Socially compliant navigation, ROS 2, LiDAR, RGBD, People tracking, Ground robot

## Abstract

We contribute a novel dataset and software tools to benchmark ROS 2 people tracking modules for ground robots. We consider a scenario where the robot moves, in a socially compliant manner, among people, tracking them accurately using multiple diverse sensors: two RGBD cameras and a planar LiDAR. The sequences recorded in the dataset are varied (in terms, e.g., of people density, people behaviors, presence of obstacles, and robot movements) to cover a wide set of application scenarios; their multi-dimensional description helps users to select appropriate subsets of sequences and to assess in which scenarios trackers manifest criticalities. The software tools provide an automated pipeline to compute tracking-relevant metrics on the dataset.

Specifications Table

**Table:** 

Subject	Computer Sciences
Specific subject area	This dataset supports the development of socially aware ground robots, providing a practical benchmark for multi-people multi-sensor people trackers.
Type of data	• Raw data stored in ROS 2 bag files with separate topics for ○ sensor data (2 RGBD cameras and one planar LiDAR scanner) ○ poses of the ground robot and the people recorded by a motion capture system • Tabular data with the description of each recorded scene.
Data collection	We recorded multiple sequences in which a ground robot navigates among people within a 6 m × 6 m area inside a controlled laboratory environment. The robotic platform mimics a commercial smart wheelchair equipped with one planar LiDAR scanner and two RGBD cameras. For each sequence, in addition to raw sensor data, the ground truth 6D poses of the robot and all people present in the scene were recorded with sub-millimeter precision using a motion capture system.
Data source location	Data was collected at the IDSIA Robotics Lab in Lugano, Switzerland.
Data accessibility	Repository name: Zenodo Data identification number: https://doi.org/10.5281/zenodo.20044662 Direct URL to data: https://zenodo.org/records/20044662 Software Tools: https://doi.org/10.5281/zenodo.20629385 The dataset is publicly available and can be accessed directly via the provided URL.
Related research article	none

## 1 Value of the Data

• The sensor configuration of the ground robot used to collect data reflects a realistic deployment scenario of a service or transportation robot. The setup is inspired by a smart wheelchair platform designed for safe autonomous navigation in indoor environments shared with people.
• The robot motion explicitly accounts for the presence and movement of surrounding people. This behavior avoids socially undesirable situations, such as crossing a person’s path or passing through groups engaged in conversation.
• The dataset supports researchers and developers in testing people tracking algorithms, particularly in the early stages of development. The recorded sequences cover a variety of typical scenarios, which help identify potential weaknesses of the tracker, including single/multiple people, stationary/dynamic people behaviors (standing, walking, running), and low-density/cluttered environments.
• Ground truth trajectories are very accurately recorded by a commercial motion capture system, avoiding errors from manual annotation.
• The dataset is distributed together with open-source software tools, detailed documentation, and usage examples, facilitating straightforward reuse for benchmarking and evaluating people tracking methods. The dataset and the tools are compatible with ROS 2, the de facto standard in robotics research.

## 2 Background

The dataset was compiled in response to limitations identified in existing datasets for robot exteroception across automotive, robotics, and social navigation domains. Automotive datasets such as H3D [1] and Waymo [2] provide rich multi-sensory data and detailed annotations but are restricted to outdoor road environments, limiting their applicability to indoor human-robot interaction scenarios. Robotics datasets often focus on tasks like object detection and 3D reconstruction, yet many rely solely on RGB imagery [[3], [4], [5], [6], [7]], making them unsuitable for platforms equipped with heterogeneous sensors such as LiDARs and RGBD cameras. Social navigation datasets capture human presence in shared environments but present gaps in annotation detail [8], human-aware interaction [9], sensing diversity [10], or variability of scene complexity [11,12]. Compared to existing datasets, this data article provides a combination of multi-sensory input, controlled variability, and precise annotations tailored to indoor social navigation scenarios. It also provides tools to benchmark custom people trackers, leveraging *TrackEval* [13], a widely used library for tracker evaluation.

## 3 Data Description

### 3.1 Sequences

The *sequences* folder contains 23 subfolders, one for each recorded scene. Folders are named with the id of the scene they contain: *s1*, …, *s23*. The data of each sequence consists of a ROS 2 bag file with the 6D poses of the robot and the people recorded by the motion capture system, the pose of the sensors, and the raw readings of the LiDAR and of the two RGBD cameras. The dataset has a total duration of 22 min and uses 75 GB of storage (32 GB for the compressed archive). The bag files contain topics published by the ROS 2 drivers of the LiDAR and those of the RGBD cameras, both at 15 Hz

**Table:** 

/camera_<i>/color/camera_info	sensor_msgs/msg/CameraInfo	RGB intrinsic parameters of camera i = 1, 2
/camera_<i>/color/image_raw	sensor_msgs/msg/Image	RGB images of camera i = 1, 2
/camera_<i>/depth/camera_info	sensor_msgs/msg/CameraInfo	Depth intrinsic parameters of camera i = 1, 2
/camera_<i>/depth/image_rect_raw	sensor_msgs/msg/Image	Depth data from camera i = 1, 2
/camera_<i>/gyro/sample	sensor_msgs/msg/Imu	IMU of camera i = 1, 2
/camera_<i>/accel/sample	sensor_msgs/msg/Imu	Accelerometer of camera i = 1, 2
/scan	sensor_msgs/msg/LaserScan	Laser scans

and topics containing the poses of the robot, the sensors and the people, published by the motion capture system at 30 Hz, in which people are assigned distinct frame ids (person_1, person_2, …)

**Table:** 

/tf_static	tf2_msgs/msg/TFMessage	Pose of sensors with respect to the robot
/tf	tf2_msgs/msg/TFMessage	Pose of robot and people in world-fixed frame

The 23 scenes present different challenges for a people tracker. In the simplest scenes, the platform does not move, and only one person is present. In other scenes, the platform is moving along with people, e.g., back-and-forth between two locations. In the most complex scenes, people are performing different behaviors/movements like standing, crouching to pick up objects, walking, running, sitting, or talking around a table.

The *sequences-test* folder contains a single short sequence, part of *s2*, to test the software pipeline without downloading the whole dataset.

### 3.2 Description

The *sequences-desc.pdf* document includes tables describing each scene along the following dimensions

**Table:** 

Duration	duration of the recording
People involved	number of people involved
Environment	s*quare* (no obstacles), c*orridor* (limited portion of the room), o*ffice* (among tables and chairs)
People behavior	*independent path* (people follow predefined individual paths), o*ne group* (people move as a single group), s*ome groups* (people form several groups), q*ueue* (people form a single queue*), multiple queues* (people form multiple queues)
People pose	*upright* (walking or standing), s*eated* (on a chair), c*rouched* (while walking, people sometimes grab objects from the floor)
People speed	*not moving, walking, or running*
People distance	*close* (people are close to the wheelchair), *mid*, or *far*
Wheelchair dynamic	*not moving, moving*
Lighting conditions	*artificial* (artificial light), *natural* (from windows)
Navigation Scenario	*frontal approach, following human*, c*rowd navigation, parallel traffic, robot crowding, not applicable* (if the wheelchair is not moving). Refer to [8] for a detailed description of the classification of navigation scenarios in human-robot interaction research.

### 3.3 Ground truth track files

As part of the evaluation of people trackers described in the next section, we extract and synchronize the poses of people in the robot frame, storing the results in tabular form in the folder *evaluation-data/mot_challenge/people_tracker-test* in the software repository.^1^ While this process is performed on the fly for poses estimated by a tracker, it can be performed just once for ground truth poses.

## 4 Experimental Design, Materials and Methods

### 4.1 Environment

We collected the dataset in an indoor room measuring 10 m x 10 m equipped with a motion capture system by Optitrack composed of 18 IR cameras, which tracks the 6D pose of rigid objects inside a 6 m x 6 m x 2 m volume, at up to 200 Hz with sub-millimeter accuracy. To track objects, we placed reflective markers on the robotic platform and on hats that people wear during recording.

The room has both artificial lights installed on the ceiling and natural light from large windows located on *one* side and equipped with curtains.

**Fig. 1:**
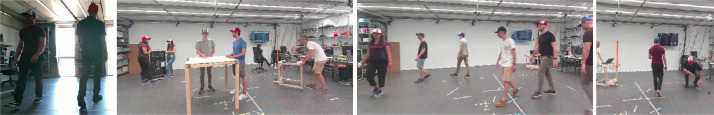
Snapshots from different scenes in the dataset.

When curtains are closed, illumination is homogeneous; otherwise, it is much higher when facing the windows, creating challenging scenarios for RGB cameras for two reasons: the strong incoming light from the windows (see Fig. 1) and the variation in illumination when the robotic platform rotates between window-facing and non-window-facing orientations. For each sequence, curtains are either open or closed for the entire duration.

### 4.2 Platform

**Fig. 2:**
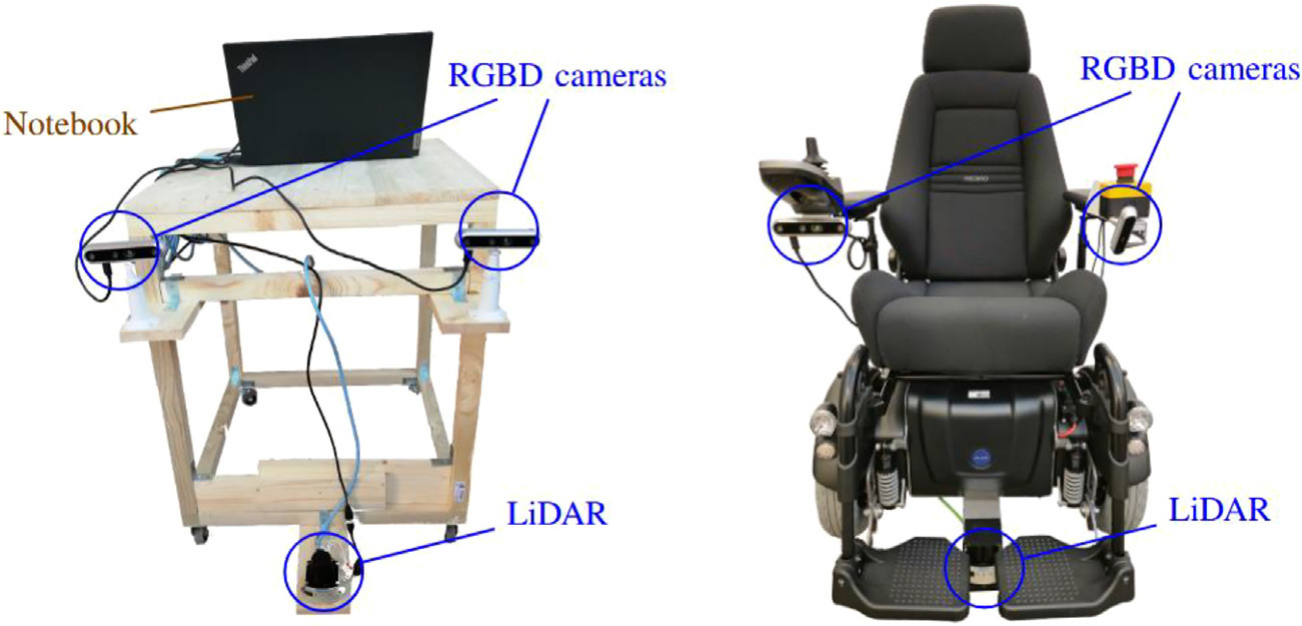
Platform. Left: Mobile platform carrying the sensors and laptop for recording the dataset. Right: Motorized wheelchair equipped with the same sensors used as a reference system (picture courtesy of BAAL labs, DFKI, Bremen).

We built the rigid mobile platform depicted in Fig. 2, which we also refer to as “robot” and “robotic platform” in the rest of the text, to carry a laptop and the sensors during the recording. Although the platform is not motorized and is manually pushed, for the purpose of recording perception data, it effectively fulfills the role of a social-aware autonomous ground robot without significant losses of realism.

The platform replicates the sensors of the smart wheelchair developed in the REXASI-PRO project^2^:

• SICK TIM571–2050101 laser scanner, controlled by the official ROS 2 driver^3^ placed below the footrest, with a resolution of 0.33° per sample and an almost symmetric field of view (limited by the platform/wheelchair) of 236°;
• two Intel RealSense D455 RGBD cameras placed on the armrests and controlled by their official ROS 2 driver.^4^

Sensors are connected via Ethernet and USB cables to a laptop running their ROS 2 drivers, configured to output data at 15 Hz.

The platform has four wheels that reproduce the motorized wheelchair kinematics with castor rear wheels and motorized front wheels. During recordings, a human operator pushes the platform around: this setup simplifies the recording and guarantees that the platform follows socially compliant trajectories required by the targeted robotics application in a social environment.

### 4.3 Subjects

A total of 12 subjects of age 25 - 45, of both genders, were involved in the recordings. They signed an informed consent form and agreed to release the recorded data for scientific purposes. The subjects were randomly assigned to the sequences. Before each sequence, subjects were instructed on the behavior to follow and are equipped with a hat with reflective markers to track them. The group of people remains the same for the recording of a sequence, which features at most 6 subjects.

### 4.4 Software tools

**Fig. 3:**
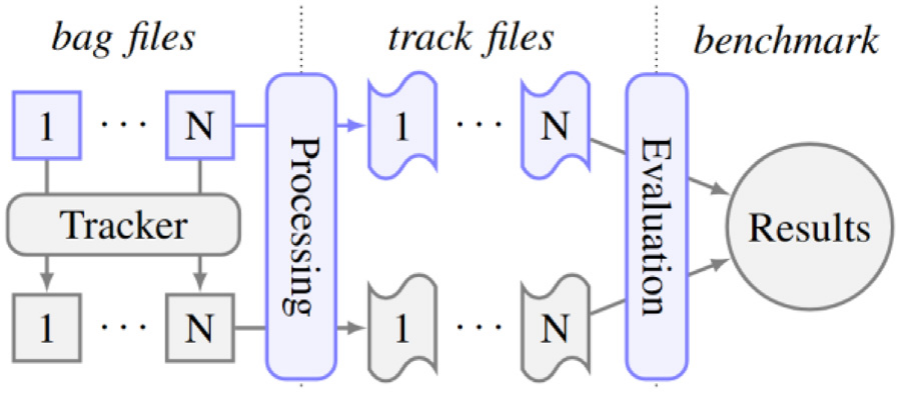
Benchmarking pipeline. Left: We record bag files with sensor readings and ground truth, which users play back to generate estimations from a tracking algorithm. Center: We extract ground truth tracks from the bag files and prepare the tracker output for evaluation. Right: We measure the tracking performance by comparing estimated tracks with the ground truth tracks. The label of bag and track files refers to different sequences (N = 23). Data and tools that we provide (including ground truth) are blue, while the tracking algorithm and data generated while benchmarking it are gray.

Alongside the dataset, we provide software tools [14] to evaluate the performance of ROS 2-compatible people trackers on the dataset. Before performing an evaluation, as a preparation step, users need to encapsulate one or more trackers in a Docker image, and modify the Docker Compose provided as a template. Then, they evaluate the trackers, performing the following automated steps, which are also illustrated in Fig. 3 and documented in the repository:

1) Run the trackers on all the sequences, storing estimated poses in new bag files.
2) Extract track files from bag files (see below), in a process that supports trackers working at arbitrary rates. The resulting estimated tracks are synchronized with the ground truth using linear interpolation and subdivided to support trackers without re-identification capability.
3) Compute multi-object tracking metrics (e.g., HOTA [15], CLEAR-MOT [16] and IDF1 [17]) using both sets of tracks (blue with ground truth and gray with estimations to *TrackEval* [13]). Results are saved in tabular form.

### 4.5 Extraction of track files

The bag files contain the poses of all people in the scene, even when they are not visible to the platform's sensors. To evaluate people trackers, we need to divide these trajectories into *tracks* that satisfy the following requirements:

• timestamps are numbered sequentially,
• no data point when people are not visible by at least one sensor,
• no data point when the motion capture loses track of any object (which may happen because of occlusions when people/platform are too close),
• different ID each time a person is out of the sensors’ field for a while (6 consecutive frames) to support trackers without long-term re-identification capability,
• detections are associated with 0.5 m wide boxes to support metrics based on intersections over unions (IoU).

**Fig. 4:**
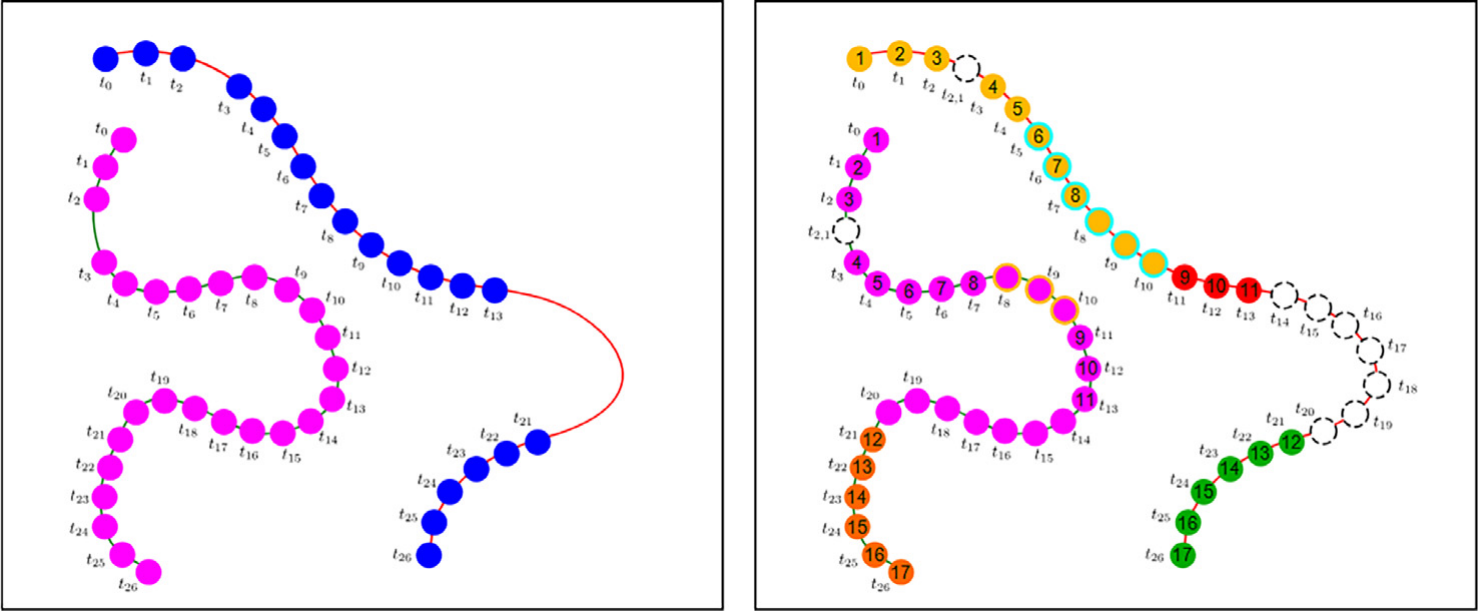
Tracks. Left: Original ground truth positions from motion capture with some missing frames for two persons (violet and blue). Right: Resulting tracks with missing (dashed) and out-of-view (cyan) frames removed, subdivided, and re-indexed.

We export tracks to a textual file format supported by *TrackEval* [13]: a table with the ID of the track, the timestamp, the position, and the (box) size. Fig. 4 illustrates the process of extracting tracks from bag files. The *doc/processing.pdf* file in the software repository, includes a track file associated with the ground truth (blue in Fig. 3) for each sequence and a detailed description of the procedure.

### 4.6 Example

The software repository contains a self-contained example (see the *README.md*) that showcases how to benchmark two particular people trackers, including a description of the trackers and an analysis of the results that provides insight into the relative strengths of the two trackers.

## Limitations

Our benchmark primarily targets the early phases of development of people trackers. We suggest complementing tests of mature trackers with more complex scenes, e.g., from JRDB [11].

The operator pushing the robotic platform was aware of the possibility of introducing artifacts due to walking. They tried their best to move smoothly, turn gently, and avoid any sudden acceleration. In the context of benchmarking people trackers, we deem the resulting motion disparity relative to a robot with motorized wheels to be negligible.

## Ethics Statement

Twelve volunteers participated in the study, with no gender-based selection criteria applied. All participants provided written informed consent, approved by SUPSI, and agreed to the use of recorded data for scientific purposes. The study complied with institutional ethical standards and the Swiss Federal Act on Data Protection, which is recognized by the European Union as providing an adequate level of data protection under the General Data Protection Regulation.

## CRediT Author Statement

**Roberto Larcher:** Conceptualization, Methodology, Software, Investigation, Writing – Original Draft; **Davide Farina:** Software, Data Curation; **Marco Piazzola:** Investigation; **Franca Corradini:** Investigation; **Carlo Grigioni:** Investigation, Validation; **Jérôme Guzzi:** Conceptualization, Methodology, Software, Writing – Review & Editing.

## Data Availability

ZenodoBenchmarking Multi-Sensor Multi-People Tracking for Ground Robots (Original data).

## Footnotes

3 https://github.com/SICKAG/sick_scan_xd.

4 https://github.com/IntelRealSense/realsense-ros.

## Footnotes

1 https://doi.org/10.5281/zenodo.20629385.

2 https://rexasi-pro.spindoxlabs.com/.

## References

[1] A. Patil, S. Malla, H. Gang, Y.-T. Chen, The H3D dataset for full-surround 3D multi-object detection and tracking in crowded urban scenes, ArXiv [Cs.CV] (2019). 10.48550/arXiv.1903.01568.

[2] Sun P., Kretzschmar H., Dotiwalla X., Chouard A., Patnaik V., Tsui P., Guo J., Zhou Y., Chai Y., Caine B., Vasudevan V., Han W., Ngiam J., Zhao H., Timofeev A., Ettinger S., Krivokon M., Gao A., Joshi A., Zhang Y., Shlens J., Chen Z., Anguelov D. (2020). 2020 IEEE/CVF Conference on Computer Vision and Pattern Recognition (CVPR).

[3] Everingham M., Van Gool L., Williams C.K.I., Winn J., Zisserman A. (2010). The pascal visual object classes (VOC) challenge. Int. J. Comput. Vis..

[4] T.-Y. Lin, M. Maire, S. Belongie, L. Bourdev, R. Girshick, J. Hays, P. Perona, D. Ramanan, C.L. Zitnick, P. Dollár, Microsoft C.O.C.O.: Common objects in context, ArXiv [Cs.CV] (2014). 10.48550/arXiv.1405.0312.

[5] O. Russakovsky, J. Deng, H. Su, J. Krause, S. Satheesh, S. Ma, Z. Huang, A. Karpathy, A. Khosla, M. Bernstein, A.C. Berg, L. Fei-Fei, ImageNet large scale visual recognition Challenge, ArXiv [Cs.CV] (2014). 10.48550/arXiv.1409.0575.

[6] M. Cordts, M. Omran, S. Ramos, T. Rehfeld, M. Enzweiler, R. Benenson, U. Franke, S. Roth, B. Schiele, The Cityscapes dataset for semantic urban scene understanding, ArXiv [Cs.CV] (2016). 10.48550/arXiv.1604.01685.

[7] A. Milan, L. Leal-Taixe, I. Reid, S. Roth, K. Schindler, MOT16: a benchmark for multi-object tracking, ArXiv [Cs.CV] (2016). 10.48550/arXiv.1603.00831.

[8] Francis A., Pérez-D’Arpino C., Li C., Xia F., Alahi A., Alami R., Bera A., Biswas A., Biswas J., Chandra R., Chiang H.-T.L., Everett M., Ha S., Hart J., How J.P., Karnan H., Lee T.-W.E., Manso L.J., Mirsky R., Pirk S., Singamaneni P.T., Stone P., Taylor A.V., Trautman P., Tsoi N., Vázquez M., Xiao X., Xu P., Yokoyama N., Toshev A., Martín-Martín R. (2025). Principles and guidelines for evaluating social robot navigation algorithms. ACM Trans. Hum. Robot Interact..

[9] H. Karnan, A. Nair, X. Xiao, G. Warnell, S. Pirk, A. Toshev, J. Hart, J. Biswas, P. Stone, Socially CompliAnt navigation dataset (SCAND): a large-scale dataset of demonstrations for social navigation, ArXiv [Cs.RO] (2022). 10.48550/arXiv.2203.15041.

[10] Álvarez-Aparicio C., Guerrero-Higueras Á.M., Olivera M.C.C., Rodríguez-Lera F.J., Martín F., Matellán V. (2017). Benchmark dataset for evaluation of range-based people tracker classifiers in mobile robots. Front. Neurorobot..

[11] Martin-Martin R., Patel M., Rezatofighi H., Shenoi A., Gwak J., Frankel E., Sadeghian A., Savarese S. (2023). JRDB: a dataset and benchmark of egocentric robot visual perception of humans in built environments. IEEE Trans. Pattern Anal. Mach. Intell..

[12] E. Vendrow, D.T. Le, J. Cai, H. Rezatofighi, JRDB-pose: a large-scale dataset for multi-person pose estimation and tracking, ArXiv [Cs.CV] (2022). 10.48550/arXiv.2210.11940.

[13] TrackEval: HOTA (and other) evaluation metrics for multi-object tracking (MOT), GitHub, n.d. https://github.com/JonathonLuiten/TrackEval (accessed May 5, 2026).

[14] Msmpt-tools, Github, n.d. https://github.com/spindoxlabs/msmpt-tools (accessed May 5, 2026).

[15] Luiten J., Os Ep A.A., Dendorfer P., Torr P., Geiger A., Leal-Taixé L., Leibe B. (2021). HOTA: a higher order metric for evaluating multi-object tracking. Int. J. Comput. Vis..

[16] Bernardin K., Stiefelhagen R. (2008). Evaluating multiple object tracking performance: the CLEAR MOT metrics. EURASIP J. Image Video Proc.

[17] E. Ristani, F. Solera, R.S. Zou, R. Cucchiara, C. Tomasi, Performance measures and a data set for multi-target, multi-camera tracking, ArXiv [Cs.CV] (2016). 10.48550/arXiv.1609.01775.

